# Supplementary material for: Early-Life Metabolic and Hormonal Markers in Blood and Growth until Age 2 Years: Results from a Randomized Controlled Trial in Healthy Infants Fed a Modified Low-Protein Infant Formula
Source: Nutrients. 2021 Apr 1;13(4):1159. doi: 10.3390/nu13041159 (PMC8067012; doi:10.3390/nu13041159)
Supplement: Supplementary file 1 [file nutrients-13-01159-s001.pdf]

Kouwenhoven et al.

Supplementary Materials: The following are available online at www.mdpi.com/xxx/s1

Table S1. Metabolic and hormonal markers at 4 months in relation to growth and body composition (measured with ADP only) through the age of 2 years.

Metabolic and hormonal markers	Age at body composition measurement	Fat mass. g	95%CI	*P*	Fat free mass. g	95%CI	*P*	Fat mass. %	95%CI	*P*
Glucose at 4 months										
	4 months	156.27	-144.64, 457.18	0.31	43.12	-202.09, 288.33	0.73	1.71	-0.71, 4.14	0.17
	6 months	92.50	-210.35, 395.34	0.55	178.13	-68.60, 424.85	0.16	0.32	-2.12, 2.76	0.80
	2 years	-91.92	-470.22, 286.38	0.63	319.29	13.20, 625.39	**0.04**	-0.95	-3.99, 2.09	0.54
Insulin at 4 months										
	4 months	9.45	-13.50, 32.39	0.42	-4.14	-22.93, 14.65	0.66	0.14	-0.04, 0.33	0.13
	6 months	16.62	-6.33, 39.57	0.16	4.03	-14.77, 22.82	0.67	0.16	-0.03, 0.34	0.10
	2 years	24.17	-3.73, 52.07	0.09	-31.69	-54.34, -9.05	**0.01**	0.23	0.01, 0.46	<u>**0.04***</u>
HOMA-IR at 4 months										
	4 months	40.37	-58.31, 139.04	0.42	-14.15	-94.98, 66.69	0.73	0.60	-0.20, 1.39	0.14
	6 months	69.69	-29.07, 168.46	0.17	20.15	-60.75, 101.05	0.62	0.62	-0.16, 1.39	0.12
	2 years	92.71	-23.55, 208.96	0.12	-123.16	-217.70, -28.63	**0.01**	0.91	-0.02, 1.84	0.06
Leptin at 4 months										
	4 months	40.74	8.06, 73.42	**0.01**	31.96	5.15, 58.77	**0.02**	0.31	0.05, 0.57	**0.02**
	6 months	46.46	13.96, 78.97	**0.01**	30.50	3.83, 57.17	**0.03**	0.33	0.07, 0.58	**0.01**
	2 years	52.78	13.83, 91.73	**0.01**	19.51	-12.21, 51.22	0.23	0.28	-0.02, 0.59	0.07
IGF-1 at 4 months										
	4 months	1.02	-1.83, 3.87	0.48	0.65	-1.70, 3.00	0.59	0.01	-0.01, 0.03	0.45

	6 months	-0.04	-2.89, 2.82	0.98	1.10	-1.25, 3.46	0.36	-0.005	-0.03, 0.02	0.68
	2 years	2.39	-1.58, 6.37	0.24	-0.45	-3.67, 2.78	0.78	0.02	-0.01, 0.05	0.26
IGF-BP1 at 4 months										
	4 months	-3.17	-10.19, 3.85	0.37	-4.70	-10.42, 1.03	0.11	-0.02	-0.07, 0.04	0.59
	6 months	-2.18	-9.18, 4.83	0.54	-9.79	-15.51, -4.08	**<0.001**	0.02	-0.04, 0.07	0.52
	2 years	-15.91	-25.21, -6.61	**<0.001**	-0.96	-8.49, 6.57	0.80	-0.12	-0.19, -0.04	**0.002**
IGF-BP2 at 4 months										
	4 months	-0.27	-0.88, 0.33	0.37	-0.55	-1.03, -0.06	**0.03**	-0.001	-0.01, 0.004	0.80
	6 months	-0.40	-1.01, 0.21	0.19	-0.88	-1.37, -0.40	**<0.001**	-0.001	-0.01, 0.004	0.73
	2 years	-0.03	-0.85, 0.79	0.94	-0.39	-1.04, 0.26	0.23	0.002	-0.005, 0.01	0.63
IGF-BP3 at 4 months										
	4 months	0.20	-0.01, 0.41	0.06	0.19	0.02, 0.35	**0.03**	0.002	-0.0001, 0.003	0.06
	6 months	0.23	0.03, 0.43	**0.03**	0.31	0.15, 0.47	**<0.001**	0.001	-0.0004, 0.003	0.14
	2 years	-0.28	-0.56, 0.00	0.05	0.26	0.03, 0.49	**0.03**	-0.002	-0.004, 0.0002	0.07

Values are associations between metabolic and hormonal markers and growth and body composition using linear mixed model analysis adjusted for feeding group and sex. BF, breast-fed; CTRL, control formula; mLP, modified low-protein formula. * Different outcome (level of significance) between original Table (Table 5).
